# Preparation and Characterization of Superfine Longan Powder Rich in Polysaccharides: Optimization of Processing Conditions and Functional Properties

**DOI:** 10.1002/fsn3.70570

**Published:** 2025-07-13

**Authors:** Jiameng Liu, Chong Chen, Zihan Wang, Lijing Lin

**Affiliations:** ^1^ Key Laboratory of Tropical Crop Products Processing of Ministry of Agriculture and Rural Affairs, Chinese Academy of Tropical Agricultural Sciences Agricultural Products Processing Research Institute Zhanjiang Guangdong China; ^2^ Hainan Key Laboratory of Storage and Processing of Fruits and Vegetables Zhanjiang Guangdong China; ^3^ College of Food Science and Technology, Guangdong Provincial Key Laboratory of Aquatic Product Processing and Safety, Guangdong Province Engineering Laboratory for Marine Biological Products Guangdong Ocean University Zhanjiang China

**Keywords:** characterization, Longan, particle size, polysaccharides, superfine grinding

## Abstract

To prepare superfine longan powder (SLP) rich in polysaccharides without adding extra ingredients, the production condition was optimized via ethanol pretreatment and response surface methodology, and the characteristics of SLP were investigated in contrast with those of the coarse longan powder. The optimal condition was determined to be: the solid–liquid ratio of 1:2, soaking time of 48 h, and ethanol concentration of 75.80%. The results indicated that superfine grinding could reduce particle size and raise the specific surface area of the powders and generate better solubility and dispersibility, but worse flowability and water‐holding capacity. Scanning electron microscopy analysis showed that large, uneven particles with visible edges changed to irregular and uniform granules after superfine grinding, and there were no new compositions and chemical groups. The results suggested that ethanol pretreatment combined with superfine grinding is an effective method to solve the technical bottleneck of longan powder produce.

## Graphical abstract

Superfine longan powder (particle size < 20 μm) rich in polysaccharides was acquired. Superfine grinding raised specific surface areas and generated better solubility. Large, uneven particles with visible edges changed to irregular and uniform granules.

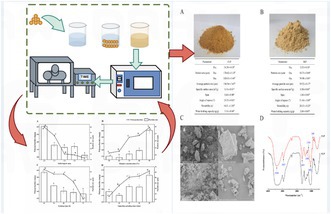

## 1 Introduction

Longan ( *Dimocarpus longan* Lour.), belonging to the Sapindaceae family, is distributed widely in the south of China (Pan et al. 2008; Jiang et al. 2013). The health benefits of longan were recorded in The Compendium of Materia Medica (Ben Cao Gang Mu in Chinese) by Li Shizhen, a famous traditional Chinese medicine expert in the Ming Dynasty, who considered longan fruit as a tonic and called it “the king of fruits” (Yi et al. 2011). The longan pulp is rich in polysaccharides, which play a critical role in the growth, development, and functional regulation of organisms and are widely involved in cell differentiation and proliferation, cell adhesion, and cellular immune defense (Xie et al. 2016). In recent years, due to the significant biological activities, such as anticoagulant activities, antitumor, antiviral, regulation of glucose and lipid metabolism, prebiotic, and immunomodulation activities, polysaccharides from natural sources have attracted much attention in the fields of pharmacology, biochemistry, and food science (Khan et al. 2019; Ferreira et al. 2015; Martins et al. 2017; Rong et al. 2019). Numerous studies have reported the medicinal value and health‐promoting mechanism of polysaccharides in longan (Lan et al. 2021; Gan et al. 2021; Yi et al. 2012). However, due to pericarp browning, fruit decay, pulp softening, and pulp breakdown, longan is rapidly perishable after harvest and during storage, resulting in the loss of nutrients (such as polysaccharides) and nutritional value (Lin et al. 2019, 2020). Several processing methods, such as drying, refrigerating, freezing, canning, and fermentation, have been implemented by manufacturers to preserve and add value to this seasonal fruit (Tippayawong et al. 2008).

Recently, the demand for fruit and vegetable powders has grown rapidly due to their great performance in flavor, color, texture, content, packing, and shipment. Besides, these powders are widely used as ingredients or functional additives in beverages and food for improving their nutritional value (Sun et al. 2019; He et al. 2019; Zhao et al. 2010). Superfine grinding has been developed as a new technology, which consumes less energy to produce micro‐sized superfine powders, enhancing characteristics that the raw material does not possess, including solubility, dispersion, surface effects, chemical reactivity, and optical properties (Wu et al. 2012). Thus, the edibility, utilization, and quality of agricultural resources can be greatly enhanced on both the sensory and nutritional sides.

Sugar constitutes more than 16% of the component in longan pulp, and its sticky behavior is attributed to the low molecular weight part, making it easy for the longan powder to absorb moisture and agglomerate during the process of preparation, which limits the superfine grinding processing (Adhikari et al. 2004). From our previous research, we found that longan pulp could be successfully prepared into ultra‐fine powder after pretreatment by soaking in ethanol solution. In this study, the pretreatment conditions, including the solid–liquid ratio, ethanol concentration, soaking time, and superfine grinding time, were optimized by response surface methodology (RSM), with the polysaccharides content and mean particle size to be taken as response variables. Besides, the effects of preparation conditions on the characteristics of longan powder were elucidated. The research results of this work could provide a reference for solving the technical bottleneck of the longan processing industry.

## 2 Materials and Methods

### 2.1 Materials and Chemicals

Dried longan ( *Dimocarpus longan* Lour.) cv. Shixia was bought from a local market. Except for food‐grade ethanol, other chemicals and solvents were of analytical grade.

### 2.2 Preparation of the Superfine Longan Powder (SLP)

Dried longan was soaked in ethanol solution at a specific ratio of solid–liquid, and the soaking solution was changed every 12 h. After that, the sample was rinsed with distilled water to remove residual ethanol from the surface, followed by grinding with a refiner, and dried at 50°C for 24 h, and then placed for 24 h. The dried sample was crushed in a universal grinder for 3 min to produce the coarse longan powder (CLP), and the SLP was obtained by superfine grinding of the CLP using a Bailey micro‐powder machine (WZJ‐6 J, China) at −15°C. CLP, as the control, was based on its representation of conventional longan powder used in both the food industry and research. By comparing the superfine longan powder (SLP) with CLP, we can observe the changes in the physical and chemical properties that occur as a result of superfine grinding. These comparisons help to highlight the effects of superfine grinding.

### 2.3 Polysaccharides Quantification

The phenol‐sulfuric acid method was based on the method of Dubois et al. (1956). The detailed procedure was as follows: (1) The sample (0.50 g) was mixed with 50 mL of distilled water and 15 mL of concentrated hydrochloric acid in a boiling water bath for 1 h and then diluted to 250 mL. (2) The obtained mixture solution (1 mL) was then mixed with 5 mL of concentrated sulfuric acid and 1 mL of 5% phenol in a test tube for 10 min. (3) The absorbance at 490 nm was measured using a spectrophotometer (UV −1780, Shimadzu, Japan), and the carbohydrate concentration was determined according to a standard curve plotted based on glucose.

### 2.4 Particle Size Determination

The mean particle size was determined using Malvern Mastersizer 2000 (Malvern Instrument Ltd., Malvern, UK). The sample was suspended in deionized water before the measurement. Measurements were done in triplicate to obtain the mean diameter. D_10_, D_50_, and D_90_ represent 10%, 50%, and 90% cumulative percentiles of the particle diameters that are smaller than the size indicated, respectively (Meng et al. 2017). The span value (span = (D_90_−D_10_)/D_50_) is used to characterize particle size distribution (Xiao et al. 2017).

### 2.5 Single‐Factor Experiments

The effect of every single factor on the polysaccharides content and mean particle size of the SLP was investigated by single‐factor experiments. Four single factors were tested and listed as follows: the solid–liquid ratio of soaking (1:2, 1:3, 1:4, 1:5, and 1:6), the ethanol concentration of soaking solution (50%, 60%, 70%, 80%, and 90%), the soaking time (24, 36, 48, 60, and 72 h), and the superfine grinding time (5, 10, 15, 20, and 25 min). In each experiment, one factor was changed within the defined range, while other factors remained unchanged.

### 2.6 Experimental Design and Optimization by RSM

Three major factors were selected for RSM analysis according to the results of single‐factor experiments. Box–Behnken design (BBD) was used to determine the optimum processing conditions for the preparation of the SLP. Based on principles of BBD, the solid–liquid ratio of soaking (*X*_1_, g/g, dried longan: soaking solution), the soaking time (*X*_2_, h), and the ethanol concentration of soaking solution (*X*_3_, %, v/v, ethanol: water) were chosen as independent design variables. The polysaccharides content (*Y*_1_, mg/g) and mean particle size (*Y*_2_, μm) in the SLP were taken as response variables of the designed experiment. Every designed parameter was studied at three different coded levels (−1, 0, 1), and their actual values were indicated in Table 1. Experimental data were obtained to perform quadratic multiple regression fitting using Design Expert 8.0.6 software (Stat‐Ease, USA). The relationship between the independent design variables and the response results was shown in Equation (1).

(1) $$Y=A_{0}+\sum_{i=1}^{3}A_{i}X_{i}+\sum_{i=1}^{3}A_{\mathrm{ii}}X_{i}^{2}+\sum_{i=1}^{2}\sum_{j=i+1}^{3}A_{\mathrm{ij}}X_{i}X_{j}+E$$

where *Y* is the predicted response result; *A*_0_, *A*_i_, *A*_ii_, and *A*_ij_ are the intercept, linear, quadratic, and interactive coefficients, respectively; *X*_i_ and *X*_j_ are the coded independent design variables; and *E* is the error term. The analysis of variance (ANOVA) was carried out to determine the significant difference in the preparation of the SLP under different operating conditions. The second‐order multiple regression equation will be used for selecting the optimum conditions for the preparation of the SLP.

**TABLE 1:** Box–Behnken experimental design and results of the response surface methodology^a^.

Run	*X*_1_: the solid–liquid ratio	*X*_2_: the soaking time (h)	*X*_3_: the ethanol concentration (%)	*Y*_1_: Polysaccharides content (mg/g)	*Y*_2_: Particle size (μm)
1	0 (1:3)	0 (48)	0 (80)	373.70	13.25
2	−1 (1:2)	1 (60)	0	350.95	17.85
3	−1	0	1 (90)	409.46	43.63
4	0	−1 (36)	−1 (70)	307.18	20.63
5	0	−1	1	376.36	47.63
6	0	0	0	371.56	22.43
7	−1	−1	0	417.40	36.62
8	1 (1:4)	0	1	336.36	16.63
9	0	0	0	380.11	19.03
10	1	−1	0	312.96	22.63
11	0	0	0	377.69	15.60
12	0	1	−1	320.48	20.08
13	0	1	1	311.00	29.36
14	1	0	−1	334.26	18.50
15	0	0	0	373.05	15.00
16	1	1	0	309.90	16.63
17	−1	0	−1	386.77	19.95

^*a*^ *X*_1_, *X*_2_, and *X*_3_ are independent variables. −1, 0, and 1 are coded values of the independent variables, corresponding to their actual values in parentheses. *Y*_1_ and *Y*_2_ are response variables.

### 2.7 Angle of Repose Determination

Firstly, a triangular glass funnel was fixed vertically above a horizontally placed glass plane, and the distance (*H*) from the glass plane to the outlet of the funnel was 1 cm. The samples of the CLP and SLP were poured into the funnel until the tip of the powder cone formed on the plane exactly touched the outlet of the funnel. The radius (*R*) of the powder cone was measured, and the angle of repose (*α*) was calculated as follows:

(2) $$\alpha \left({}^{\circ}\right)=\arctan\frac{H}{R}$$

### 2.8 Wettability Determination

The samples of the CLP and SLP (1.00 g) were placed individually in beakers containing 25 mL of distilled water at 25°C. The time (s) required for the sample to get wet was recorded. Each test was performed three times, and the average value was determined.

### 2.9 Water‐Holding Capacity (WHC) Determination

The water‐holding capacity (WHC) of the CLP and SLP was measured following the descriptions of He et al. (2019). Firstly, the samples (*M*_1_) and the empty centrifuge tubes (*M*_2_) were weighed separately. Then, 50 mL of water and the samples were mixed in the centrifuge tubes and stirred for 30 min under 25°C, followed by centrifugation for 30 min at 2000 rpm. The supernatant was removed and then the wet samples, together with the tubes (*M*_3_), were measured. The WHC of the samples was calculated using the following formula.

(3) $$\mathrm{WHC}\;\left(g/g\right)=\frac{M_{3}-M_{2}-M_{1}}{M_{1}}$$

### 2.10 Scanning Electron Microscopy (SEM)

Surface morphology of the CLP and SLP was studied using a scanning electron microscope (S‐4800; HITACHI, Tokyo, Japan) at an operating voltage of 10 kV. Samples were fixed onto the sticky film, and excess materials were removed using an air compressor. Then, samples were coated with gold using an ion sputter coater and observed at a magnification of 100×, 500×, and 1000×.

### 2.11 Fourier Transform Infrared Spectroscopy (FTIR)

The samples of CLP and SLP were analyzed using a Fourier transform infrared spectrophotometer (FTIR spectrometer, PerkinElmer) according to the method of Zhao et al. (2015). Spectra were obtained in the wavelength range from 4000 to 400 cm^−1^, and 32 scans at a resolution of 4 cm^−1^ were applied to each test sample.

### 2.12 Statistical Analysis

The experimental results were presented as “means ± standard deviations” of at least triplicate independent determinations. Data were analyzed by IBM SPSS Statistics 21.0 software (SPSS Inc., Chicago, IL, USA), and the significant difference was determined at *p* < 0.01 or *p* < 0.05 by Duncan's multiple range test.

## 3 Results and Discussion

### 3.1 Optimization of Preparation Process of the SLP

#### 3.1.1 Results of Single‐Factor Experiments

**FIGURE 1:**
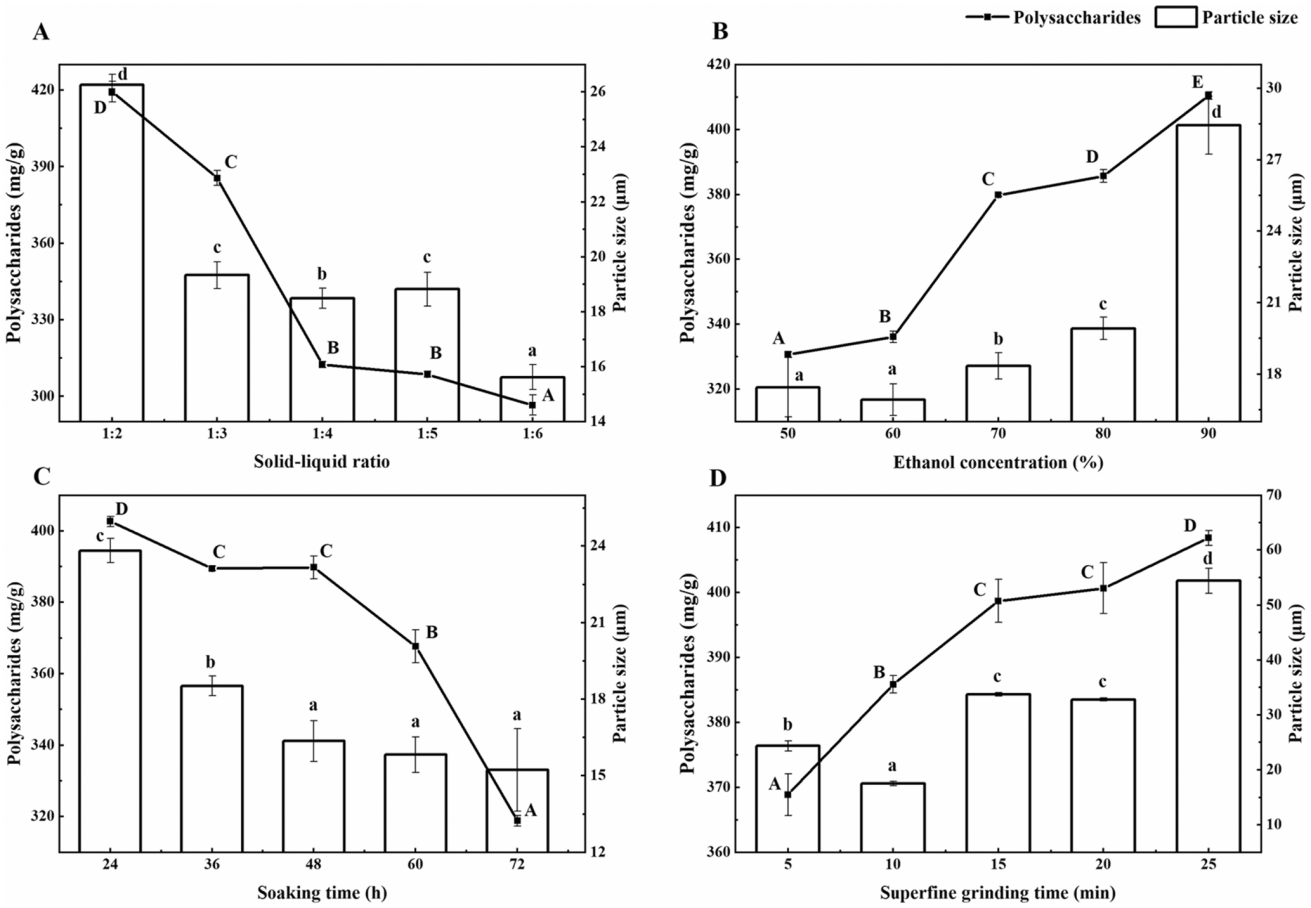
The polysaccharides content and mean particle size of the superfine longan powder (SLP) in single‐factor experiments under different solid–liquid ratios (A), ethanol concentrations (B), soaking times (C), and superfine grinding times (D). Different lowercase letters indicate significant differences (*n* = 3, *p* < 0.05) among the data for the particle size of samples. Different uppercase letters indicate significant differences (*n* = 3, *p* < 0.05) among the data for the polysaccharides content of samples.

According to Figure 1A, the content of polysaccharides in samples decreased significantly when the solid–liquid ratio changed from 1:2 to 1:4 (the content of polysaccharides changed from 419.40 mg/g to 312.42 mg/g) (*p* < 0.05), followed by a slow reduction while the solid–liquid ratio changed to 1:6 (the content of polysaccharides reached 296.56 mg/g). The particle size decreased significantly when the solid–liquid ratio changed from 1:2 to 1:4 (the particle size changed from 26.27 μm to 18.49 μm) and from 1:5 to 1:6 (the particle size changed from 18.83 μm to 15.63 μm) but remained stable at other times. When the solid–liquid ratio was 1:3, the particle size (19.34 μm) reached the level of superfine powder (< 20 μm); thus, “1:3” was confirmed as the optimum solid–liquid ratio (Zhao et al. 2015).

It can be seen from Figure 1B that with the increase of the ethanol concentration, there has been a significant and continued increase in the content of polysaccharides in samples from 330.74 mg/g to 410.42 mg/g (*p* < 0.05). While the particle size showed a gradual increase (from 17.45 to 19.92 μm) before the ethanol concentration reached 80% and then dramatically increased to 28.45 μm. Therefore, “80%” was chosen as the optimal ethanol concentration at which the sample contained 385.69 mg/g of polysaccharides and 19.92 μm of particle size.

Figure 1C shows that with the increase of the soaking time, the content of polysaccharides in samples decreased gradually from 402.67 mg/g to 389.75 mg/g within 48 h but significantly to 318.82 mg/g thereafter (*p* < 0.05). Besides, the particle size declined significantly from 23.83 μm to 16.36 μm before soaking the sample for 48 h (*p* < 0.05) and then remained largely the same. “48 h” was chosen as the most suitable soaking time.

Figure 1D shows the impact of superfine grinding time on both the polysaccharides content and the particle size. The content of polysaccharides in samples increased significantly when superfine grinding for 5 to 15 min and 20 to 25 min (*p* < 0.05), with the lowest value being 368.88 mg/g and the highest 408.40 mg/g, reflecting that the grinding time has little effect on the polysaccharides content compared to the other three single factors. Regarding the particle size, an overall growth trend is observed, along with a significant gap between the maximum and minimum values. The minimum particle size (17.53 μm) was achieved when the sample was ground for 10 min. Consequently, the superfine grinding time was fixed at 10 min.

#### 3.1.2 Analysis of the Response Surfaces

RSM was used to analyze the interactions of all variables and provide optimal conditions in the tested range. The experimental model of process optimization by RSM was based on the former results of single‐factor experiments. The designed model and the results are shown in Table 1, based on which the quadratic multiple regression fitting was performed by Design Expert 8.0.6 software. The quadratic polynomial equations Equation (4) and Equation (5) (listed as follows), along with their ANOVA (shown in Table 2), were obtained. It can be seen from Table 2 that both the model of the polysaccharides content (*Y*_1_, *R* ^2^ = 0.9858) and particle size (*Y*_2_, *R* ^2^ = 0.9353) were significant (*p* < 0.05), while their lack of fit was not significant (*p* > 0.05), indicating that the quadratic model is suitable for this experiment.

(4) $$Y_{1}=375.22-33.89X_{1}-15.20X_{2}+10.56X_{3}+15.85X_{1}X_{2}-5.15X_{1}X_{3}-19.67X_{2}X_{3}+5.27{X_{1}}^{2}-32.69{X_{2}}^{2}-13.78{X_{3}}^{2}$$

(5) $$Y_{2}=17.06-5.46X_{1}-5.45X_{2}+7.26X_{3}+3.19X_{1}X_{2}-6.39X_{1}X_{3}-4.43X_{2}X_{3}+0.81{X_{1}}^{2}+5.56{X_{2}}^{2}+6.80{X_{3}}^{2}$$

**TABLE 2:** Analysis of variance (ANOVA) for the fitted quadratic polynomial model of the polysaccharides content and particle size^a^.

Response variables	Source	Model	*X*_1_	*X*_2_	*X*_3_	*X*_1_ *X*_2_	*X*_1_ *X*_3_	*X*_2_ *X*_3_	*X*_1_^2^	*X*_2_^2^	*X*_3_^2^	Residual	Lack of fit	Pure error	Cor total
*Y*_1_: Polysaccharides content (mg/g)	Sum of squares	20128.36	9187.44	1847.35	892.45	1004.51	105.88	1546.85	116.99	4498.95	799.4	290.26	239.86	50.4	20418.62
	DF	9	1	1	1	1	1	1	1	1	1	7	3	4	16
	Mean square	2236.48	9187.44	1847.35	892.45	1004.51	105.88	1546.85	116.99	4498.95	799.4	41.47	79.95	12.6	
	*F*	53.93	221.56	44.55	21.52	24.22	2.55	37.3	2.82	108.5	19.28		6.35		
	*p*	< 0.0001	< 0.0001	0.0003	0.0024	0.0017	0.1541	0.0005	0.1369	< 0.0001	0.0032		0.0531		
	Prob > F	**	**	**	**	**		**		**	**				
*Y*_2_: Particle size (μm)	Sum of squares	1532.47	238.27	237.51	421.95	40.77	163.2	78.5	2.79	130.1	194.91	106.08	52.55	53.54	1638.55
	DF	9	1	1	1	1	1	1	1	1	1	7	3	4	16
	Mean square	170.27	238.27	237.51	421.95	40.77	163.2	78.5	2.79	130.1	194.91	15.15	17.52	13.38	
	*F*	11.24	15.72	15.67	27.84	2.69	10.77	5.18	0.18	8.59	12.86		1.31		
	*p*	0.0021	0.0054	0.0055	0.0012	0.145	0.0135	0.057	0.6809	0.022	0.0089		0.3875		
	Prob > F	**	**	**	**		*			*	**				

^*a*^ *X*_1_, *X*_2_, and *X*_3_ are independent variables: *X*_1_ represents the solid–liquid ratio, *X*_2_ represents the soaking time, and *X*_3_ represents the ethanol concentration.

* Significant at 5% (*p* < 0.05).

** Significant at 1% (*p* < 0.01); *R* ^2^(*Y*_1_) = 0.9858, *R* ^2^(*Y*_2_) = 0.9535.

**FIGURE 2:**
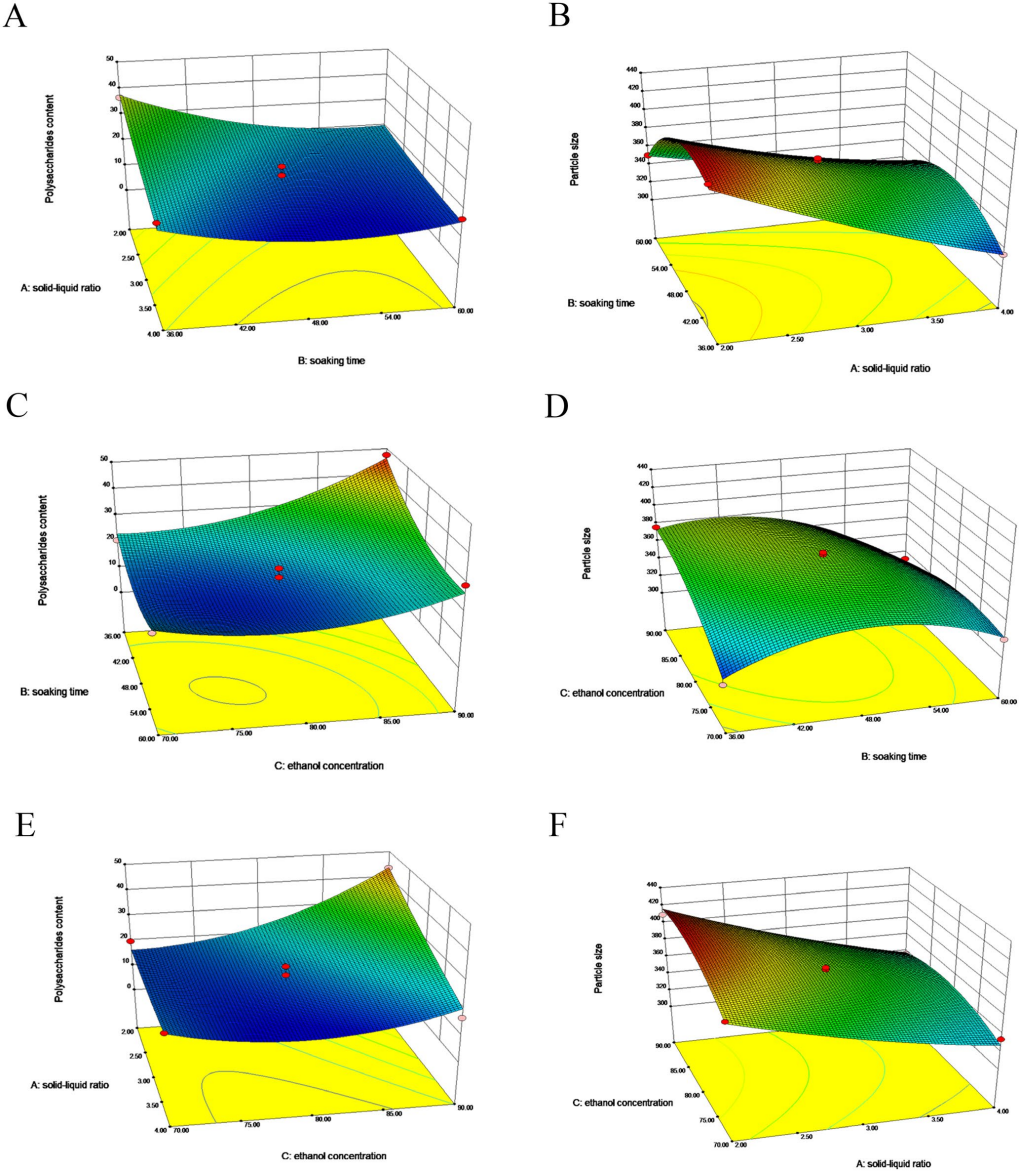
Response surface diagram for the effect of the solid–liquid ratio, soaking time, and ethanol concentration on the polysaccharides content (A, C, E) and particle size (B, D, F) in the SLP.

The three‐dimensional response surface diagram derived from the regression equation of the polysaccharides content and particle size is shown in Figure 2, reflecting the effect of interaction among the three factors *X*_1_, *X*_2_, and *X*_3_ on the response value. It can be seen from the *F* value that the influence of each factor on the polysaccharides content follows this decreasing order: Solid–liquid ratio > soaking time > ethanol concentration, while that on the particle size manifests as: Ethanol concentration > solid–liquid ratio > soaking time.

#### 3.1.3 Verification of the Response Surface Model

The ideal condition for the SLP preparation optimized by Design Expert software was as follows: the solid–liquid ratio was 1:2, soaking time was 48.03 h, and ethanol concentration was 75.83%, while the polysaccharides content and particle size in SLP were 405.39 mg/g and 18.81 μm, respectively, in the model. To facilitate actual production, conditions were adjusted as follows: the solid–liquid ratio was 1:2, soaking time was 48 h, and ethanol concentration was 75.80%. Under optimal conditions, the actual experiment was conducted. The results showed that the polysaccharides content (*Y*_1_) in the SLP was 398.69 mg/g, and the particle size (*Y*_2_) was 19.52 μm, with deviations of 1.65% and 3.77% compared to the predicted values, indicating that the optimized preparation scheme is reasonable and effective.

### 3.2 Characterization of Longan Powder

#### 3.2.1 Particle Size Distribution

**FIGURE 3:**
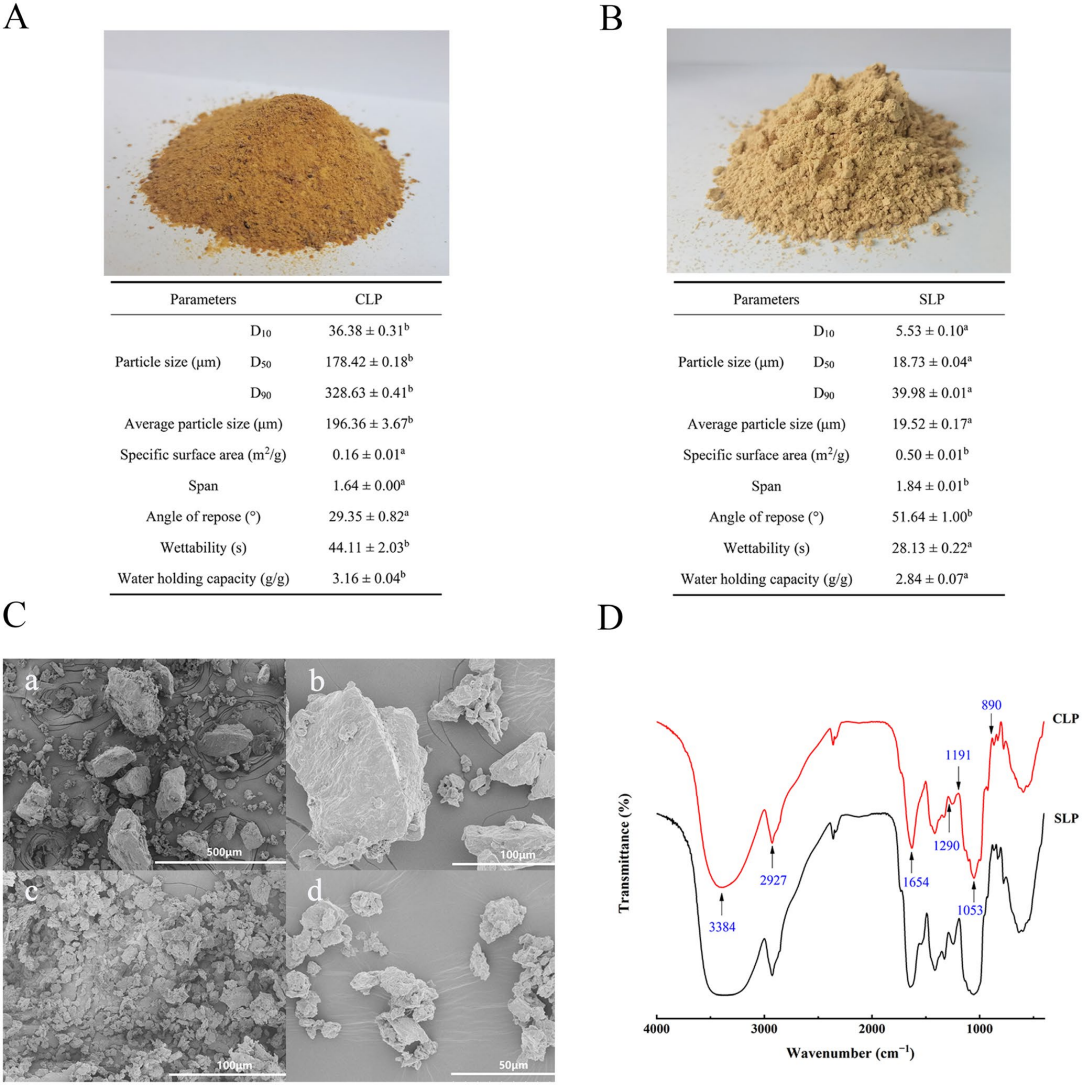
Characterization of the coarse longan powder (CLP) and SLP: Micromeritic parameters and powder properties of the CLP (A) and SLP (B); D10, D50, and D90 represent 10%, 50%, and 90% cumulative percentiles of the particle diameters that are smaller than the size indicated, respectively; Means of the same parameters of CLP and SLP with different lowercase letters differ significantly (*n* = 3, *p* < 0.05). Surface morphology of CLP (a: 100×, b: 500×) and SLP (c: 500×, d: 1000×) (C). FTIR spectra of CLP and SLP (D).

The particle size distribution of the CLP and SLP was investigated and presented in Figure 3A,B. D_50_ values were 178.42 and 18.73 μm, respectively, differing by nearly 10 times. The average particle size of the CLP was 196.36 μm and that decreased significantly to 19.52 μm in the SLP, while the specific surface area increased by more than twofold. The results indicated that superfine grinding could reduce powder particle size and raise specific surface area, causing improvement in the bioavailability and absorption (Ramachandraiah and Chin 2016). Besides, the particle size distribution (span values) in the SLP was slightly wider than that in the CLP, and this was in accordance with the results of He et al. (2019).

#### 3.2.2 Powder Properties

Figure 3A,B also show some of the basic properties of the CLP and SLP. The angle of repose increased significantly from 29.35° to 51.64° with a decrease in particle size, indicating that the flowability of the powders became worse after grinding (Ileleji and Zhou 2008). The result was not in agreement with the investigation of Zhao et al. (2010) and Sun et al. (2019). One possible explanation is that the longan powder is rich in polysaccharides, which are cohesive materials, and the exposure of longan polysaccharides to the air during superfine grinding may cause a reduction in repose angle, resulting in a decrease in fluidity (Zhao et al. 2015). Wettability is one of the most important indexes to evaluate the solubility of powders (Li et al. 2020). As shown in Figure 3A,B, the wetting suspension time of the SLP was significantly less than that of the CLP (*p* < 0.05), indicating that the superfine powder shows better solubility and dispersibility than the coarse powder, which is consistent with the findings of Li et al. (2020). The WHC of the SLP (2.84 g/g) was significantly lower than that of the CLP (3.16 g/g). In general, since the increase in specific surface area after superfine grinding, the WHC should increase as reported in Zhao et al. (2010). However, other research has reported that coarse powders have higher WHC because of larger spaces than superfine powders, suggesting that there might be other interfering factors involved (Ramachandraiah and Chin 2016).

#### 3.2.3 SEM Analysis

As can be seen from Figure 3C, the CLP particles displayed as some large, uneven blocks, and the edges of the particles were obvious. A small part has a particle size of up to 200–300 μm, while most of the particles were about 100 μm. However, the SLP consisted of irregular and uniform granules, indicating that superfine grinding made the longan powder into smaller fractions and resulted in various shapes (Zhao et al. 2015). The results were consistent with the particle size measurement in Figure 3A,B. Superfine grinding altered the structure of longan powder particles; thus, it could cause changes in their physical–chemical properties (Zhao et al. 2010).

#### 3.2.4 FTIR Spectroscopy

**FIGURE 4:**
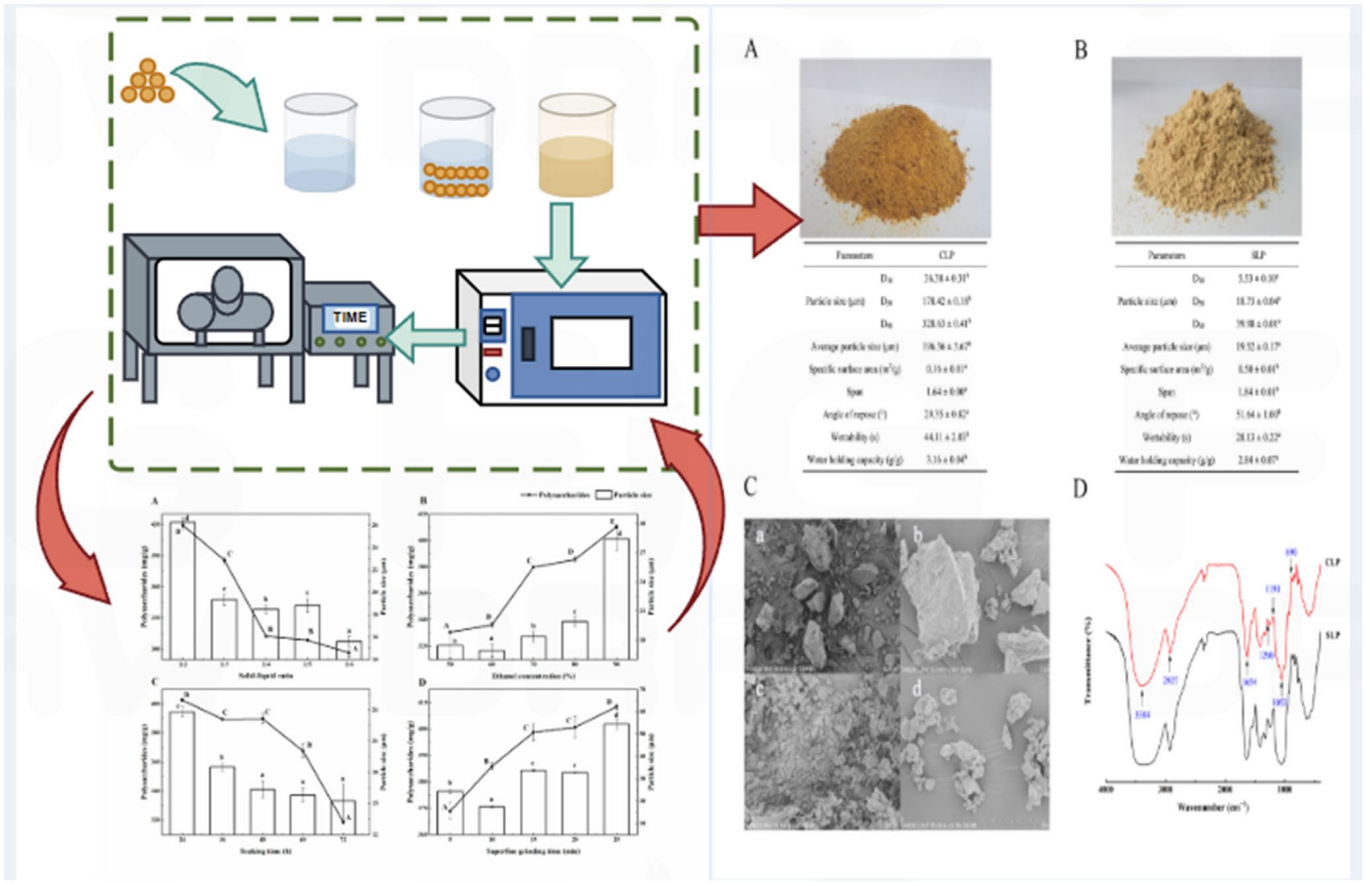
Graphical table of contents. (By Figdraw.)

FTIR spectra of the CLP and SLP are shown in Figure 3D. In general, the broad band at 3500–3200 cm^−1^ and signals at 2927 and 1290 cm^−1^ were attributed to the O‐H stretching vibration, C‐H stretching vibration, and C‐H bending vibration, respectively, as characteristic absorption bands of polysaccharides (Zhu, Dong, et al. 2016). Protein structures can also be indicated by the absorption around 1645 cm^−1^, suggesting the presence of proteins in the CLP and SLP (Liao et al. 2015). The absorption bands at 1191 and 1053 cm^−1^ were characteristic absorptions of stretching vibrations of ester carbonyl groups (C‐O‐C) of the *α*‐pyranose ring in the glucosyl residue (Zhu, Luo, et al. 2016). The signals around 890 cm^−1^ were characteristic absorptions of *α*‐type glycosidic linkage bending vibrations and D‐glucopyranose ring vibrations (Chen et al. 2016). The spectrum of the SLP resembled that of the CLP, indicating that there no new composition, and chemical groups were produced in the superfine grinding procedure (Figure 4).

## 4 Conclusion

In this study, BBD and RSM were effectively used to estimate the effect of the solid–liquid ratio, ethanol concentration, soaking time, and their interactions on the polysaccharides content and mean particle size of the SLP, as well as to determine the optimal processing condition to produce the SLP: the solid–liquid ratio was 1:2, soaking time was 48 h, and ethanol concentration was 75.80%. It was confirmed to be reasonable and effective via a confirmatory experiment, with the polysaccharides content and particle size to be 398.69 mg/g and 19.52 μm, respectively, in SLP. The results of the powder characteristics measurement indicated that superfine grinding could reduce powder particle size and raise specific surface area, thus causing an improvement in the bioavailability and absorption. The superfine powder shows better solubility and dispersibility than the coarse powder, but worse flowability and lower WHC, and it may result from the cohesive characteristics of the polysaccharides in longan powder. The images of the SLP and CLP indicated that large, uneven particles with visible edges changed to irregular and uniform granules in the processing of superfine grinding, and there were no new compositions and chemical groups produced according to FTIR spectra. This work makes it possible to prepare ultra‐fine longan powder and retain longan polysaccharides without adding extra ingredients.

The SLP demonstrates promising potential as a functional ingredient in the food industry. Due to its smaller particle size and higher surface area, the SLP exhibits enhanced solubility and dispersibility, making it suitable for incorporation into various food formulations. In addition, the presence of bioactive polysaccharides in the SLP suggests its potential use in the development of functional foods with health‐promoting properties, such as immune‐modulating, anti‐inflammatory, and antioxidative effects. These properties, along with their ability to improve the texture, nutritional value, and sensory qualities of food products, open up a wide range of applications in food products such as beverages, dietary supplements, and functional convenience foods. Furthermore, the improved water‐holding capacity and solubility of the SLP could make it beneficial in the preparation of low‐fat, high‐fiber food items. Thus, the superfine grinding process not only enhances the physical properties of longan powder but also significantly broadens its scope of use in food‐related industries.

## Author Contributions

**Jiameng Liu:** formal analysis (lead), investigation (equal), methodology (equal), software (lead), visualization (lead), writing – original draft (lead). **Chong Chen:** data curation (equal), validation (lead), writing – review and editing (lead). **Zihan Wang:** data curation (equal), investigation (equal), methodology (equal). **Lijing Lin:** funding acquisition (lead), project administration (lead), resources (lead).

## Conflicts of Interest

The authors declare no conflicts of interest.

## Data Availability

The data that support the findings of this study are available from the corresponding author upon reasonable request.
